# Soluble Form of Receptor for Advanced Glycation End Products Is Associated with Obesity and Metabolic Syndrome in Adolescents

**DOI:** 10.1155/2014/657607

**Published:** 2014-01-20

**Authors:** Chih-Tsueng He, Chien-Hsing Lee, Chang-Hsun Hsieh, Fone-Ching Hsiao, Philip Kuo, Nain-Feng Chu, Yi-Jen Hung

**Affiliations:** ^1^Department of Internal Medicine, Tri-Service General Hospital Songshan Branch, Taipei 10581, Taiwan; ^2^Division of Endocrinology and Metabolism, Department of Internal Medicine, Tri-Service General Hospital, National Defense Medical Center, No. 325, Section 2, Chenggong Road, Neihu District, Taipei City 11490, Taiwan; ^3^College of Medicine, University of Illinois at Chicago, Chicago, IL 60612, USA; ^4^School of Public Health, National Defense Medical Center, No. 161, Section 6, Minquan E. Road, Neihu District, Taipei City 11490, Taiwan

## Abstract

The aim of this cross-sectional study was to investigate the relationship between soluble form of receptor for advanced glycation end products (sRAGE), obesity, and metabolic syndrome (MetS) in adolescents. A total of 522 male and 561 female adolescents were enrolled into the final analyses. Anthropometric parameters, blood pressure, blood biochemistry, fasting insulin, and plasma sRAGE levels were measured. In males, sRAGE was significantly and inversely correlated with waist circumference (WC), body mass index (BMI), systolic blood pressure, triglyceride (TG), low density lipoprotein cholesterol (LDL-C), and homeostasis model assessment-insulin resistance (HOMA-IR). Only WC and BMI were significantly and inversely correlated with sRAGE in females. Using linear regression analysis adjusting for age and gender, significant association was found between sRAGE and WC, BMI, TG, LDL-C, and HOMA-IR in adolescents of either gender (*P* < 0.05). This association was abolished when further adjusting BMI. In addition, sRAGE was significantly and inversely correlated with the increasing number of components of MetS in males (*P* for trend = 0.006) but not in females (*P* for trend = 0.422). In conclusion, plasma sRAGE is associated with obesity and MetS among adolescents. BMI may be the most important determinant of sRAGE levels in adolescents.

## 1. Introduction

The metabolic syndrome (MetS), which includes abdominal obesity, dyslipidemia, hypertension, and hyperglycemia, all documented risk factors for cardiovascular disease (CVD), has become one of the major public health challenges in developed and developing countries. The increasing prevalence of the syndrome is associated with the global epidemic of obesity and diabetes [1]. The International Diabetes Federation (IDF) identifies central obesity, measured by waist circumference, as an obligatory finding for the diagnosis of MetS [2]. The causes of the MetS are multifactorial, stemming from complex genetic and environmental influences. Insulin resistance is believed to play a major role in the underlying pathophysiology of the MetS, possibly contributing to atherosclerosis and increasing the risk of glucose intolerance and diabetes [3]. However, the cellular and molecular bases for the pathologic phenomena occurring in the MetS have not been fully elucidated.

Advanced glycation end products (AGE) are formed from proteins and peptides by nonenzymatic glycoxidation after contact with aldose sugars [4, 5]. AGEs are known to accumulate in the vessel wall, where they may act to alter the extracellular matrix, cell surface receptors, or the function of intracellular proteins [6]. Interactions between AGE and its receptors (RAGE) elicit oxidative stress and induce proinflammatory or procoagulant cellular responses including increases in the vascular cell adhesion molecule-1 and tumor necrosis factor-alpha expression [7]. These responses are thought to be mediated by the activation of nuclear factor-*κ*B (NF-*κ*B) and eventually accelerate the process of atherosclerosis.

The RAGE is a multiligand receptor of the immunoglobulin superfamily that engages diverse ligands relevant to the pathogenesis of atherosclerosis [8]. RAGE has a C-truncated secreted isoform, termed soluble RAGE (sRAGE). In contrast to the cell surface RAGE, sRAGE blocks cell surface RAGE-ligand binding and subsequent signaling by acting as a decoy [9]. Recent studies have reported reduced levels of sRAGE as an important novel biomarker in patients with hypertension and type 2 diabetes and in nondiabetic subjects with coronary artery disease [10–12]. Some studies have also demonstrated that levels of plasma sRAGE are inversely correlated with the components of the MetS—systemic arterial pressure, body mass index (BMI), waist-to-hip ratio, serum triglycerides (TG), and insulin resistance index—in the adult population [13, 14]. Accordingly, sRAGE, obesity, and MetS are strongly associated with atherosclerosis. The process of atherosclerosis begins during adolescence or even earlier [15]. However, few studies have explored the relationship between sRAGE, obesity, and MetS among adolescents and past studies have yielded inconsistent results. Hence, our study enrolled a larger population to investigate the relationship between plasma sRAGE, obesity, and components of MetS among junior high school adolescents in Taiwan.

## 2. Materials and Methods

### 2.1. Study Design and Sampling

The Taipei Children Heart Study-III was an epidemiological study conducted in 2006 investigating obesity and other risk factors of CVD among school adolescents in Taipei City, Taiwan. In order to obtain a representative distribution of demographic and lifestyle characteristics, we conducted a cross-sectional survey among junior high school students in Taipei City. After a multistage sampling of 16 junior high schools, 1500 school adolescents were randomly selected for this survey. The sampling method and results are described elsewhere [16, 17]. After taking into account study power and excluding missing data, a total of 1083 adolescents (522 males and 561 females) with the mean age of 13.7 years (from 12 to 16) were included in the final analyses.

### 2.2. Data Collection

The Ethical Committee of the Scientific Institute approved this study and informed consent was obtained from the participant adolescents and their parents. All participating adolescents completed a structured questionnaire detailing their sociodemographic characteristics, personal history of disease, and pubertal development.

### 2.3. Anthropometric Measurements

Research technicians measured body weight to an accuracy of 0.1 kg using a standard beam balance scale with subjects barefoot and wearing light indoor clothing. Body height was recorded to the nearest 0.5 cm using a ruler attached to the scale. We calculated their BMI as body weight (kg) divided by the square of their height (m). Waist circumference (WC) was measured to the nearest 0.1 cm at the midpoint between the inferior margin of the last rib and the iliac crest.

### 2.4. Blood Pressure

After 10-minute rest, we measured blood pressure (BP) in a sitting position on the right arm using an appropriate cuff size; the first and fifth Korotkoff sounds were recorded as systolic BP (SBP) and diastolic BP (DBP). We measured BP again after a 5-minute rest period and the average of the two measurements was used for analysis.

### 2.5. Biochemical Measurements

We collected blood samples after a 10-hour fast and only from students who had followed their usual dietary pattern during the previous 3 days to reduce extraneous intersubject variation. Any students who had recently attended a holiday feast or family party were recontacted several weeks later. Plasma glucose concentrations were analyzed immediately after blood sampling; biochemical assays of lipid profiles were performed within 2 weeks of the blood samples being stored at −4°C. Plasma insulin levels were measured with a commercial immunoradiometric kit (BioSource Europe, Nivelles, Belgium). The intra- and interassay coefficients of variation (CV) for insulin were 2.2% and 6.5%, respectively. Plasma glucose concentrations were determined by the glucose oxidase method on a Beckman Glucose Analyzer II (Beckman Instruments, Fullerton, California). The intra- and interassay CV for glucose were 0.6% and 1.5%, respectively. Serum levels of total cholesterol (TC), TG, and low density lipoprotein cholesterol (LDL-C) were measured using the dry, multilayer analytical slide method in the Fuji Dri-Chem 3000 analyzer (Fuji Photo Film, Tokyo, Japan). The intra- and interassay CV for LDL-C were 0.8% and 2.5%, respectively. Serum levels of high density lipoprotein cholesterol (HDL-C) were determined by an enzymatic cholesterol assay method after dextran sulfate precipitation. The intra- and interassay CV for HDL-C were 1.1% and 1.7%, respectively. Insulin resistance (IR) was assessed using the homeostasis model assessment model of insulin resistance (HOMA-IR) described by Matthews et al. [18]. According to the criteria of the IDF [19], the diagnosis of the MetS was made by abdominal obesity (WC ≥ 90th percentile) and the presence of two or more other clinical features (i.e., TG ≥ 150 mg/dL, HDL-C < 40 mg/dL, SBP ≥ 130 mmHg or DBP ≥ 90 mmHg, or plasma glucose ≥ 100 mg/dL) in our study population.

### 2.6. Determination of Plasma sRAGE Levels

Plasma sRAGE levels were determined using a commercial ELISA kit (Quantikine ELISA kit; R&D Systems, Minneapolis, MN, USA), containing the basic components required for the development of solid-phase sandwich ELISAs, according to the manufacturer's instructions. The intra- and interassay CV were 4.8% and 8.2%, respectively. Detection limit for the assay was 16.14 pg/mL.

### 2.7. Statistical Analyses

Mean and standard deviation (SD) were used to describe the distributions of age, body weight, body height, BMI, WC, biochemical components of MetS, HOMA-IR, and sRAGE level with gender specification. Group differences between both genders were analyzed by Student's *t*-test for continuous variables. Simple linear regression was used to calculate the clinical and anthropometric parameters by different sRAGE categories. We calculated the Spearman correlation coefficient between study variables to evaluate the associations of sRAGE on anthropometric measures, BP, and biochemical components of MetS with gender specification without the assumption of normality. Multiple linear regression models were used to calculate the association between cardiometabolic variables and sRAGE. One-way ANOVA was used to compare the means of plasma sRAGE levels between numbers of components of MetS in either gender. A two-tailed *P* value less than 0.05 was considered statistically significant. Statistical analyses were performed by using software (SAS version 9.1.3, SAS Institute, Cary, NC, USA).

## 3. Results

A total 1083 adolescents (522 males and 561 females) were included in the final analyses. The prevalence of obesity (determined by WC ≥ 90th percentile) and MetS in our study population was 19.2% and 4.1%, respectively.  Table 1 shows the clinical and anthropometric parameters of adolescents in this study according to gender. The mean age of participants was 13.7 ± 0.9 years. Male adolescents had significantly higher WC, BMI, SBP, and fasting glucose levels but lower TC, HDL-C, and LDL-C levels than female adolescents. There was no significant difference in DBP, TG, and HOMA-IR index between both genders. Plasma sRAGE levels (mean ± SD) were slightly, but insignificantly, higher in male compared to female adolescents (1423 ± 849 pg/mL versus 1343 ± 800 pg/mL; *P* = 0.11).

We further analyzed the clinical and anthropometric parameters of adolescents in this study by quartiles of plasma sRAGE levels (Table 2). In male adolescents, the WC, BMI, SBP, TG, LDL-C, and HOMA-IR levels showed a significantly inverse tendency with the quartiles of plasma sRAGE levels. The HDL-C level showed a significantly increased tendency with the quartiles of plasma sRAGE levels instead. However, in female adolescents, only the WC and BMI levels showed a significantly inverse tendency with the quartiles of plasma sRAGE levels.

Investigation of plasma sRAGE levels with cardiometabolic variables showed a significantly inverse correlation with WC, BMI, SBP, TG, LDL-C, and HOMA-IR in male adolescents. This significantly inverse correlation was only observed with WC and BMI in female adolescents (Table 3).

In the linear regression analysis, we included all the cardiometabolic parameters as independent variables (WC, BMI, SBP, DBP, fasting glucose, TG, TC, HDL-C, LDL-C, and HOMA-IR) (Table 4). Results of model I analysis show that WC, BMI, TG, HDL-C, LDL-C, and HOMA-IR significantly predicted plasma sRAGE levels after adjusting for age and gender. But further adjusting BMI in model II found no other cardiometabolic parameters that had significant power to predict the plasma sRAGE levels. BMI was implied as the most independent factor to predict the plasma sRAGE levels in adolescents.

The comparison of the means of plasma sRAGE levels between numbers of components of MetS—from zero to all five—was analyzed (Table 5). In male adolescents, the plasma sRAGE level decreased significantly when the number of components of MetS increased (*P* for trend: 0.006). In female adolescents, we observed a tendency of decreased plasma sRAGE level with an increased number of components of MetS, but this was not significant (*P* for trend: 0.422).

## 4. Discussion

To the best of our knowledge, this is the largest study investigating the association between sRAGE, obesity, and MetS, particularly in adolescents. Our study demonstrates that plasma sRAGE level is inversely correlated with MetS and its components—including obesity indices, SBP, and TG mainly in the male adolescent population. BMI was determined to be the most independent predictive factor for plasma sRAGE levels in adolescents. Our results support previous findings from a study done by Norata et al. [14], which showed that sRAGE is inversely associated with BMI and waist-to-hip ratio in the general population, mainly in adults aged 40 to 80. Their findings suggested that plasma sRAGE levels may reflect a metabolic perturbant status that could later lead to vascular complications and diabetes prior to any clinical complication. We further extended this hypothesis by focusing our study on adolescents. However, our results showed a significant relationship between plasma sRAGE levels and MetS and its components, which was inconsistent with Norata's findings. In their study, the plasma sRAGE levels between subjects with and without MetS were similar. The reason for this discrepancy is not well known. One possible explanation may be that our study selected participants with relatively healthy clinical and anthropometric parameters (lower WC, BMI, BP, glucose, TG, TC, and LDL-C levels) or perhaps differences in ethnicity and/or age attributed to this discrepancy. Another explanation for the discrepancy is different criteria for accessing the MetS. In Norata's study, the presence of the metabolic syndrome was assessed according to the National Cholesterol Education Program, Adult Treatment Panel III (NCEP-ATPIII) guidelines. The definition of the MetS proposed by IDF [19] was adopted in this study because the IDF definition is more adapted to an adolescent population as compared to the NCEP-ATPIII one which has been designed for adults in the first place.

Few studies have investigated the relationship between sRAGE and obesity in children or adolescents. A recent study by Šebeková et al., which enrolled few patients, revealed no significant association between sRAGE and obesity in children and adolescents [20], contrary to our results. However, participants in their study had higher sRAGE levels than ours. The measurement methods of sRAGE levels in Šebeková's and our studies are not different. Therefore, the inconsistence may be due to ethnic differences. However, a significantly inverse relationship between sRAGE and HOMA-IR was revealed in their study, which was similar to our results. This result also supported the findings from Basta et al. [11] that plasma level of sRAGE is downregulated in insulin resistance status.

The mechanism of interaction between sRAGE, obesity, and MetS is still unclear at present. Many ligands which interact with RAGE are important inflammatory regulators [21]. It is conceivable that inflammation may link the interaction between sRAGE, obesity, and MetS. Shoelson et al. have proposed potential cellular mechanisms for activating this inflammatory signaling [22]. In addition, another study further demonstrated that RAGE could regulate atherosclerosis through adiposity by using RAGE apoE double deficient mice [21]. Worldwide, the increasing prevalence of obesity in the recent decades is startling and is likely a cause of the rising incidence of insulin resistance and MetS, as well as CVD and type 2 diabetes. Current literature supports the notion that the presence of the MetS in youth may be an important predictor of future risk for diabetes and CVD. There is substantial evidence that obesity is the main determinant of insulin resistance in children and that it increases the risk not only for the MetS in adulthood but also for CVD and type 2 diabetes later in life [23]. A recent prospective study showed that, in adolescence, an elevated BMI—one that is well within the range currently considered to be normal—constitutes a substantial risk factor for obesity-related disorders in midlife [24]. Recently, Brix et al. [25] demonstrated a significant increase of sRAGE levels after the dramatic weight loss induced by bariatric surgery in adults with morbid obesity. Hence, our results further demonstrate the potential role of sRAGE in the pathogenesis of obesity and MetS in the earlier life.

Endogenous secretory RAGE (esRAGE) is one of the splice variants that directs the synthesis of RAGE proteins carrying all the extracellular domains but lacks the transmembrane and intracytoplasmic domains [13]. Previous studies have shown a link between esRAGE and atherosclerosis and MetS [13, 26]. However, the sample size in these studies is relatively small. In addition, few studies investigated the relationship between esRAGE, obesity, and MetS in adolescents. Therefore, we decided to measure the total sRAGE in our study.

There are some limitations in this study. First, our participants are relatively healthy adolescents in Taiwan. The results could not be applied to adults or to adolescents not living in Taiwan, even though previous studies have shown similar results in different populations with different disease statuses. In addition, a recent study conducted in nondiabetic young-to-middle-aged medication-free subjects [27] showed that sRAGE levels decline with increasing number of risk factors, prior to the manifestation of MetS, with central obesity underlying the onset. Our study confirms this finding in the adolescents, shading the light on early onset of the decline. Second, this study is of cross-sectional design. We could not predict the future incidence of disease, especially CVD in adulthood, even though many results of studies conducted in adults have supported the hypothesis that sRAGE is a potential factor against obesity, MetS, and atherosclerosis. Further longitudinal or prospective studies are needed to confirm our results. Third, pubertal stage of participants was not examined by our researchers but derived from a self-reported questionnaire, which may be prone to confounding effects. Hence, we decided to exclude the pubertal stage as a variable of this analysis. As a result, we did not know whether our results were influenced by puberty or not.

## 5. Conclusions

We have demonstrated that plasma sRAGE is associated with obesity and MetS among junior high school adolescents in Taiwan. Our findings indicate that BMI may be the most important determinant of plasma sRAGE level in adolescent. Although MetS-associated decline in sRAGE might be driven by incipient atherosclerotic changes, the role of sRAGE in the pathogenesis of adolescent obesity and MetS needs further studies to elucidate.

## Figures and Tables

**Table 1:** Clinical and anthropometric parameters of participants by gender.

	Male	Female	*P*
	(*n* = 522)	(*n* = 561)	
Age (years)	13.7 ± 0.9	13.7 ± 0.9	
Waist circumference (cm)	78.6 ± 10.7	74.0 ± 8.0	∗
BMI (kg/m^2^)	21.6 ± 4.2	20.4 ± 3.4	∗
Systolic blood pressure (mmHg)	118 ± 14	112 ± 12	∗
Diastolic blood pressure (mmHg)	69 ± 11	70 ± 9	
Fasting plasma glucose (mg/dL)	94 ± 7	92 ± 7	∗
Triglycerides (mg/dL)	71 ± 38	71 ± 30	
Total cholesterol (mg/dL)	163 ± 27	174 ± 29	∗
HDL cholesterol (mg/dL)	47 ± 11	52 ± 11	∗
LDL cholesterol (mg/dL)	92 ± 25	97 ± 24	∗
HOMA-IR	3.38 ± 2.52	3.25 ± 1.85	
sRAGE (pg/mL)	1423 ± 849	1343 ± 800	

All data is expressed as mean ± standard deviation. *P < 0.05 (comparison between genders). BMI: body mass index; HDL: high density lipoprotein; LDL: low density lipoprotein; HOMA-IR: homeostasis model assessment-insulin resistance; sRAGE: soluble form of receptor for advanced glycation end products.

**Table 2:** Clinical and anthropometric parameters by different sRAGE categories.

	Male (*n* = 522)				Female (*n* = 561)			
	Q1 sRAGE ≤ 888.9	Q2 888.9 < sRAGE ≤ 1339.3	Q3 1339.3 < sRAGE ≤ 1818.5	Q4 sRAGE > 1818.5	Q1 sRAGE ≤ 750.5	Q2 750.5 < sRAGE ≤ 1253.9	Q3 1253.9 < sRAGE ≤ 1807.0	Q4 sRAGE > 1807.0
Age (years)	13.7 ± 0.9	13.6 ± 0.9	13.8 ± 0.9	13.7 ± 0.8	13.8 ± 0.9	13.7 ± 0.8	13.7 ± 0.8	13.8 ± 0.9
WC (cm)	81.8 ± 11.3***	78.8 ± 11.1	78.8 ± 10.5	75.0 ± 8.8	75.0 ± 8.5***	75.9 ± 9.2	72.6 ± 6.6	72.3 ± 7.0
BMI (kg/m^2^)	23.1 ± 4.5***	21.8 ± 4.3	21.5 ± 4.1	20.0 ± 3.3	21.0 ± 3.5***	21.3 ± 3.7	19.7 ± 3.0	19.6 ± 3.0
SBP (mmHg)	120.2 ± 14.3*	118.0 ± 14.3	118.3 ± 13.1	116.3 ± 14.4	111.3 ± 12.1	111.0 ± 13.8	113.2 ± 11.9	111.3 ± 11.9
DBP (mmHg)	70.6 ± 10.1	68.4 ± 10.2	69.2 ± 9.6	68.7 ± 11.9	69.8 ± 9.6	69.7 ± 10.0	70.4 ± 8.8	69.5 ± 9.5
FPG (mg/dL)	94.2 ± 7.3	94.0 ± 6.9	93.6 ± 5.9	93.5 ± 6.4	91.3 ± 6.8	92.0 ± 7.1	91.3 ± 6.9	92.2 ± 7.4
TG (mg/dL)	79.8 ± 48.4**	69.2 ± 32.0	65.7 ± 34.4	67.6 ± 31.7	71.5 ± 28.1	74.4 ± 33.4	72.2 ± 33.3	67.2 ± 25.6
TC (mg/dL)	166.6 ± 27.9	163.1 ± 27.8	160.2 ± 28.1	162.3 ± 25.3	177.4 ± 30.5	170.2 ± 29.0	174.8 ± 29.5	173.5 ± 26.7
HDL-C (mg/dL)	46.2 ± 10.0*	47.0 ± 11.9	47.3 ± 11.4	49.1 ± 11.2	52.2 ± 11.9	49.2 ± 9.5	52.9 ± 11.5	52.5 ± 11.5
LDL-C (mg/dL)	96.4±28.0**	94.3 ± 25.9	89.6 ± 24.7	89.2 ± 21.4	99.4 ± 25.1	96.0 ± 26.6	95.3 ± 24.4	96.6 ± 21.4
HOMA-IR	4.0 ± 3.2***	3.5 ± 2.6	3.3 ± 2.3	2.8 ± 1.6	3.3 ± 1.8	3.4 ± 1.9	3.4 ± 2.3	2.9 ± 1.3

All data is expressed as mean ± standard deviation. Q1 to Q4 stand for quartiles. Unit of sRAGE: pg/mL. *P < 0.05, **P < 0.01, and ***P < 0.001 test for trend by gender. sRAGE: soluble form of receptor for advanced glycation end products; WC: waist circumference; BMI: body mass index; SBP: systolic blood pressure; DBP: diastolic blood pressure; FPG: fasting plasma glucose; TG: triglycerides; TC: total cholesterol; HDL-C: high density lipoprotein cholesterol; LDL-C: low density lipoprotein cholesterol; HOMA-IR: homeostasis model assessment-insulin resistance.

**Table 3:** Spearman correlation coefficient of cardiometabolic variables associated with sRAGE.

	Male (*n* = 522)	Female (*n* = 561)
	*r*	*r*
Age	0.03	−0.03
Waist circumference	−0.21***	−0.14**
BMI	−0.25***	−0.18***
Systolic blood pressure	−0.11*	0.03
Diastolic blood pressure	−0.06	<0.01
Fasting plasma glucose	−0.02	0.02
Triglycerides	−0.10*	−0.07
Total cholesterol	−0.07	−0.03
HDL cholesterol	0.08	0.05
LDL cholesterol	−0.11*	−0.04
HOMA-IR	−0.17***	−0.06

*P < 0.05, **P < 0.01, and ***P < 0.001. sRAGE: soluble form of receptor for advanced glycation end products; BMI: body mass index; HDL: high density lipoprotein; LDL: low density lipoprotein; HOMA-IR: homeostasis model assessment-insulin resistance.

**Table 4:** Linear regression of cardiometabolic variables associated with sRAGE in both genders.

	Model I^a^	Model II^b^
	*β* (S.E)	*β* (S.E)
Waist circumference	−1.6 (0.3)***	<0.1 (0.1)
BMI	−0.8 (0.1)***	—
Systolic blood pressure	−0.5 (0.4)	0.1 (0.4)
Diastolic blood pressure	−0.3 (0.3)	<0.1 (0.3)
Fasting plasma glucose	<−0.1 (0.2)	<0.1 (0.2)
Triglycerides	−2.7 (0.9)**	−0.9 (0.9)
Total cholesterol	−1.1 (0.8)	−1.0 (0.8)
HDL cholesterol	0.7 (0.3)*	−0.1 (0.3)
LDL cholesterol	−1.8 (0.7)**	−0.7 (0.7)
HOMA-IR	−0.2 (0.1)***	<−0.1 (0.1)

^a^Model I: after adjusting for age and gender. ^b^Model II: after adjusting for age, gender, and BMI. *P < 0.05, **P < 0.01, and ***P < 0.001. *β*: standardized regression coefficient; S.E: standard error; sRAGE: soluble form of receptor for advanced glycation end products; BMI: body mass index; HDL: high density lipoprotein; LDL: low density lipoprotein; HOMA-IR: homeostasis model assessment-insulin resistance.

**Table 5:** Comparison of plasma sRAGE levels between numbers of components of MetS by gender.

Numbers of components of MetS	Male		Female	
	*n*	sRAGE (pg/mL)	*n*	sRAGE (pg/mL)
0	242	1540.2 ± 891.6	321	1379.7 ± 855.0
1	169	1393.4 ± 776.3	167	1315.3 ± 738.8
2	80	1256.4 ± 900.9	55	1298.1 ± 707.7
≧3	31	1107.0 ± 563.9	18	1092.5 ± 552.4

*P* for trend		0.006		0.422

All data is expressed as mean ± standard deviation. sRAGE: soluble form of receptor for advanced glycation end products; MetS: metabolic syndrome.
